# An analysis of *Methylenetetrahydrofolate reductase *and *Glutathione S-transferase omega-1 genes *as modifiers of the cerebral response to ischemia

**DOI:** 10.1186/1471-2377-9-37

**Published:** 2009-07-22

**Authors:** Leema Reddy Peddareddygari, Ana Virginia Dutra, Mark A Levenstien, Souvik Sen, Raji P Grewal

**Affiliations:** 1 New Jersey Neuroscience Institute at JFK Medical Center, 65 James Street, Edison, New Jersey, USA; 2 Program in Cancer Biology and Genetics, Memorial Sloan-Kettering Cancer Center, 1275 York Avenue, New York, New York, USA; 3 UNC Hospital Stroke Center, 7003A Neuroscience Hospital, CB 7025, Chapel Hill, North Carolina, USA

## Abstract

**Background:**

Cerebral ischemia involves a series of reactions which ultimately influence the final volume of a brain infarction. We hypothesize that polymorphisms in genes encoding proteins involved in these reactions could act as modifiers of the cerebral response to ischemia and impact the resultant stroke volume. The final volume of a cerebral infarct is important as it correlates with the morbidity and mortality associated with non-lacunar ischemic strokes.

**Methods:**

The proteins encoded by the methylenetetrahydrofolate reductase (*MTHFR*) and glutathione S-transferase omega-1 (*GSTO-1*) genes are, through oxidative mechanisms, key participants in the cerebral response to ischemia. On the basis of these biological activities, they were selected as candidate genes for further investigation. We analyzed the C677T polymorphism in the *MTHFR *gene and the C419A polymorphism in the *GSTO-1 *gene in 128 patients with non-lacunar ischemic strokes.

**Results:**

We found no significant association of either the *MTHFR *(p = 0.72) or *GSTO-1 *(p = 0.58) polymorphisms with cerebral infarct volume.

**Conclusion:**

Our study shows no major gene effect of either the *MTHFR *or *GSTO-1 *genes as a modifier of ischemic stroke volume. However, given the relatively small sample size, a minor gene effect is not excluded by this investigation.

## Background

In the United States, after cardiovascular disease and cancer, stroke is the third leading cause of death. However, it is the leading cause of morbidity resulting in about 4 million stroke survivors each year [1]. Although there are epidemiological differences in various ethnic groups, the majority of these strokes are ischemic rather than hemorrhagic in nature. There are a number of well established risk factors for the development of ischemic stroke including diabetes mellitus, atrial fibrillation, hypertension and age [2]. Once an ischemic stroke occurs, in spite of intense research efforts, little progress has been made in the discovery or development of effective neuroprotective agents.

While there are recent research efforts investigating susceptibility genes for ischemic stroke, there are few investigations of the genetics of the cerebral response to ischemia [3,4]. Studies of induced strokes in transgenic animals indicate that manipulation of certain genes can influence the resultant volume [5,6]. We hypothesize that there is variability in the cerebral response to ischemia that is mediated by polymorphisms in genes encoding proteins which participate in this response. Polymorphisms in these genes could enhance or diminish endogenous neuroprotective mechanisms and ultimately impact the volume of an ischemic stroke. This has clinical significance because, in general, the volume of a stroke correlates with severity and the resultant degree of disability of the patient [7,8].

It has been established that cerebral ischemic injury results in a core of necrotic tissue surrounded by a penumbra of tissue in which neurons are functionally inactive but still potentially viable [9]. The development of this ischemic penumbra is limited in time by a cascade of reactions in response to the initial ischemia followed by subsequent reperfusion. Reperfusion occurring shortly after ischemia reduces infarct volume, but at a later period may exacerbate ischemic injury [10]. It has been suggested that reperfusion increases reactive oxygen species (ROS) production which can have further deleterious consequences. Neuronal cell exposure to these ROS, which include nitric oxide, superoxide ions and hydroxyl radicals, can result in oxidative deoxyribonucleic acid (DNA) damage [11-14].

**Figure 1:**
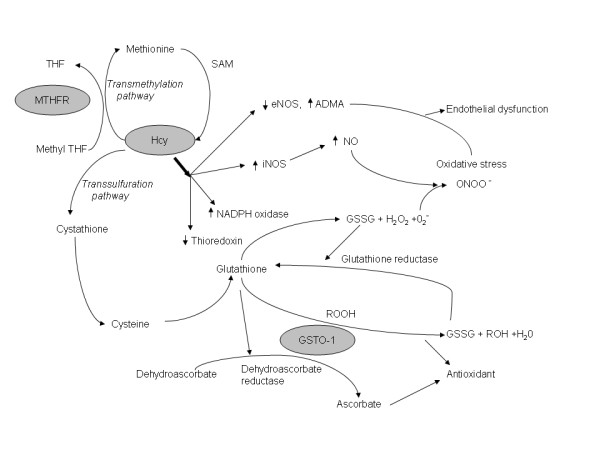
**The metabolic pathways regulating oxidative stress**. Abbreviations: MTHFR- Methylenetetrahydrofolate reductase, THF- tetrahydro folate, Hcy- Homocysteine, SAM- S-Adenosyl methionine, eNOS-endothelial nitric oxide synthase, iNOS-inducible nitric oxide synthase, ADMA- asymmetric dimethylarginine, NO- nitric oxide, O_2_^- ^– superoxide radical, ONOO^- ^peroxinitrite, H_2_O_2_-hydrogen peroxide, NADPH – nicotinamide adenine dinucleotide phosphate, GSTO-1- Glutathione S-transferase omega-1, GSSG -glutathione disulfide, ROOH- reduced hydroperoxides, ROH- alcohol, H_2_O- water.

Methylenetetrahydrofolate reductase (*MTHFR*) and Glutathione S-transferase omega-1(*GSTO-1*) are two genes that are key participants in the metabolic pathways regulating oxidative stress in the brain (Fig 1). The C677T polymorphism (NCBI SNP cluster ID rs# 1801133) in *MTHFR *gene results in an alanine-to-valine (A222V) substitution which in turn causes a reduction in enzyme activity and subsequent elevation of plasma homocysteine [15,16]. Hyperhomocysteinemia (HHcy) has been reported as an independent risk factor for stroke but may also influence ischemic stroke volume. A study of the cerebral volume and induced stroke following middle cerebral artery occlusion in mice showed that HHcy was associated with increased oxidative damage and larger ischemic lesion [17-19]. The homocysteine (Hcy) dependent trans-sulfuration pathway is responsible for maintenance of the intracellular glutathione pool and regulation of this pathway under oxidative stress (Fig 1). GSTO-1 is member of Phase II enzymes that catalyze glutathione-dependent antioxidant pathways (Fig 1). Furthermore GSTO-1 modulates rynodyne receptors thereby protecting cells against Ca^2+ ^induced apoptosis and inhibition of the posttranslational processing of pro-inflammatory cytokine interleukin-1β [20,21]. The C419A (NCBI SNP cluster ID, rs 4925) polymorphism in *GSTO-1 *gene has been reported to be involved in stroke [22]. The C419A polymorphism results in amino acid alteration alanine-to-aspartate; this change in the protein sequence reduces enzyme activity and therefore could influence tissue susceptibility to oxidative stress [23].

Given the key roles of the products of these two genes in the cerebral response to ischemia, they are candidate genes which warrant further investigation. We hypothesized that the *MTHFR *C677T and *GSTO-1 *C419A polymorphisms may modify the response of the brain to cerebral ischemia and ultimately impact the final stroke volume. To test our hypothesis we studied the relationship of these polymorphisms with infarct volume measured in patients who have suffered an ischemic stroke.

## Methods

### Human subjects

We studied 128 patients admitted to the stroke unit at John F. Kennedy Medical Center, Edison, New Jersey with non-lacunar ischemic infarcts. The strokes were categorized according to the Trial of ORG 10172 in Acute Stroke Treatment (TOAST) [24]. Among these patients, applying the TOAST classification, the etiology of the non-lacunar ischemic strokes included 37 large artery atherothrombosis, 37 cardioembolic, 49 unknown and 5 others. In the majority of patients (79%), diffusion-weighted magnetic resonance imaging scans were available for review; alternatively, cerebral computed tomography scans (21%) were used to measure stroke volume. The stroke volume was obtained by averaging the calculated values from direct measurements of the cerebral images by two examiners as previously described [25]. Informed consents were obtained from the patients or their proxies and study was conducted according to protocols and methods approved by the local Institutional Review Board.

### Genotyping

Genomic DNA was isolated from blood samples (Puregene Systems, Gentra). The *MTHFR *polymorphisms were genotyped as previously described [15]. Briefly, the primer pairs used were F: 5'-TGAAGGAGAAGGTGTCTGCGGGA-3' and R: 5'-AGGACGGTGCGGTGAGAGTG-3'. The C677T polymorphism creates a restriction site for Hinf I, so the digestion of the polymerase chain reaction (PCR) products of the mutant allele generates two fragments (175 and 23 bp). The digested products were resolved by gel electrophoresis (6% polyacrylamide gel) and the allele frequency was obtained by direct gene counting. The genotypes obtained with this method were confirmed by direct sequencing.

The *GSTO-1 *polymorphism was detected after PCR amplication using following primer pairs F: 5'-TGTCTAGGTGCCATCCTTGGT-3' and R: 5'-AAGTGACTTGGAAAGTGGGAA-3'. PCR was carried out in a final volume of 50 μl with 40 ng of DNA, 600 μM dNTP and 2.5 U Amplitaq gold polymerase. Thermal cycling consisted of initial activation (95°C for 7 min) followed by 35 cycles of denaturation (95°C, 30 s), annealing (56°C, 30 s), and extension (72°C, 30 s) and a final extention at 72°C for 7 min. PCR products were digested with restriction enzyme Cac8I at 37°C overnight and the digested products were resolved by gel electrophoresis (6% polyacrylamide gel). The digestion generates two fragments (147 bp and 60 bp) corresponding to C (restriction site present) and a single 207 bp product corresponding to the A allele (restriction site absent). The allele frequency was obtained by direct gene counting. The genotypes obtained with this method were confirmed by direct sequencing.

## Results

The clinical and demographic data of the study group is presented in table 1. The genotypes of the *MTHFR *C677T and *GSTO-1 *C419A polymorphisms are presented in table 2. The observed numbers of each genotype was compared with those expected under Hardy-Weinberg equilibrium (HWE) by using a web-based program . No significant deviation from HWE was observed. The mean Hcy levels corresponding to each genotype is presented in the table 3. Although the mean Hcy level is higher in the group with *MTHFR *TT genotype, it is not statistically significant. To detect the effect of the *MTHFR *C677T and *GSTO-1 *C419A polymorphisms on stroke volume ANOVA and generalized linear model analysis was done. The mean stroke volume was 71.37 cm^3 ^(SD 90.1 cm^3^). There was no significant influence of the *MTHFR *(p = 0.72) or *GSTO-1 *(p = 0.58) polymorphisms on the stroke volume. The analysis was also performed for Caucasian population alone which represented 67.2% of the study population. However no significant effect of either *MTHFR *(p = 0.41) or *GSTO-1 *(p = 0.83) polymorphisms on the size of stroke was detected.

**Table 1:** Patient characteristics.

Demographic factors	
Female n (%)	69 (52.4)
Age, y mean (S.D^a^)	70.7 (13.1)
Ethnicity n (%)	
Caucasians	86 (67.2)
African Americans	9 (7.0)
Hispanics	13 (10.2)
Others^b^	20 (15.6)
Co morbidities^c ^n (%)	
Hypertension	105 (82.0)
Diabetes	55 (43.0)
Atrial fibrillation	37 (28.9)
Carotid stenosis = 70%	41 (32.3)
TOAST Classification^d ^n (%)	
Large artery atherosclerosis	37 (28.9)
Cardiembolism	37 (28.9)
Unknown^e^	49 (38.3)
Others	5 (3.9)

The clinical and demographic data of the study group.

^a ^S.D = Standard deviation.

^b ^Include Asians and other ethnic groups.

^c ^Other factors studied include Coronary artery disease, Peripheral vascular disease, Lipid profile, Homocysteine levels.

^d ^TOAST = Trial of Org 10172 in Acute Stroke Treatment.

^e ^Include stroke of undetermined etiology or those due to two or more causes.

**Table 2:** Genotype and stroke volume data.

	Genotype	N	Mean stroke volume (cm^3^)
C677T	CC	49	59.5
	CT	60	84.6
	TT	18	61.5
C419A	CC	55	72.7
	AC	54	74.0
	AA	14	63.9

**Table 3:** Genotype and homocysteine data

	Genotype	N	Mean plasma homocysteine (μmol/L)
*MTHFR *C677T	CC	38	10.7
	CT	44	10.8
	TT	14	11.7
*GSTO-1 *C419A	CC	43	10.7
	AC	40	10.7
	AA	12	10.3

## Discussion

Our analysis did not show a major gene effect of either the *MTHFR *C677T polymorphism or *GSTO-1 *C419A polymorphism on the volume of the ischemic infarct. However, minor genetic effects are not excluded by this study. It is possible that such minor gene effects may be additive and particular combinations of polymorphisms from different genes may confer neuroprotection. These minor gene effects could be detected by studying a much larger sample size.

In addition to investigating genes involved in the response of the brain following ischemia, there are other variables that could confound the analysis. For example, due to our relatively small sample size we did not separate and analyze the volumes of strokes occurring in a particular vascular distribution. To enhance the detection of minor gene effects, future studies could focus only upon middle cerebral artery stroke. In this study, again due to small sample size, we did not limit our analysis to those strokes of a particular etiology but rather focused upon the entire group. There may to be biological/genetic differences in the cerebral response to ischemia in cardioembolic stroke compared with large artery atherosclerosis.

## Conclusion

In spite of the negative results, our study demonstrates the feasibility of studying the potential role of genes which participate in the cerebral response to ischemia and ultimately influence stroke volume. Many of the difficulties of any such investigation can be overcome by studying a larger sample size of patients. These studies could provide insight into endogenous neuroprotective mechanisms and facilitate the development of more effective therapies.

## Abbreviations

MTHFR: Methylenetetrahydrofolate reductase; GSTO-1: Glutathione S-transferase omega-1; ROS: Reactive oxygen species; DNA: Deoxyribonucleic acid; HHcy: Hyperhomocysteinemia; Hcy: Homocysteine; TOAST: Trial of ORG 10172 in Acute Stroke Treatment; PCR: Polymerase chain reaction; HWE: Hardy-Weinberg equilibrium.

## Competing interests

The authors declare that they have no competing interests.

## Authors' contributions

LRP carried out the molecular genetic study and analysis and drafted the manuscript. AVD assisted with the molecular genetic study. ML performed the statistical analysis. SS participated in the design and finalizing the manuscript. RG participated in the design, coordination of the study and finalized the manuscript. The manuscript is read and approved by all the authors.

## Pre-publication history

The pre-publication history for this paper can be accessed here:


